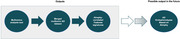# Genetics, atrophy, liquid biomarkers and cognitive tests in Alzheimer’s disease: an integrated and multimodal analysis pipeline to understand the neurodegenerative mechanisms

**DOI:** 10.1002/alz.088671

**Published:** 2025-01-09

**Authors:** Margherita Squillario, Francesco Reggiani, Matteo Pardini, Michele Piana

**Affiliations:** ^1^ IRCCS Ospedale Policlinico San Martino, Genova, Genova Italy; ^2^ Università degli studi di Genova, Genova, Liguria Italy

## Abstract

**Background:**

Alzheimer’s disease (AD) is the most common neurodegenerative disease among the elderly (50–75%) and the causes of the sporadic form are not yet clear. The genetic predisposition is relevant (60–80%), but alone is not sufficient to trigger those initial processes that lead to neurodegeneration and to the slowly atrophy of the brain. Several public datasets containing multimodal data centered on AD have emerged since the beginning of 2004 when ADNI come out. An appropriate integration of various data types (e.g., biomedical images, GWAS/WES, cognitive tests, liquor protein expression) is pivotal to understand this phenomenon.

**Methods:**

Objective 1: we will develop a merged multimodal AD‐centered dataset from a careful selection of public database. Objective 2: we will develop a user‐friendly analysis tool starting from the multikernel/deep learning pipeline devised to properly address the analysis of multimodal data. Objective 3: in the merged multimodal AD‐centered dataset we will evaluate the predictive performance of gene‐SNPs panels, retrieved from the literature, using the developed tool. We will identify clusters of genes‐SNPs (of the panel) and of specific neurodegenerative markers correlating with the atrophy (measure by FDG‐PET) of the most affected brain regions through a weighted correlation network analysis (WCNA). Objective 4: we will validate those clusters in silico, in an independent partition of the merged multimodal dataset and/or in independent AD public dataset.

**Results:**

The analysis of the merged multimodal AD dataset with the proper multimodal‐oriented tool, will identify genes‐SNPs signatures that correlate with atrophy starting from (i) promising genes‐SNPs panels identified in the literature and (ii) neurodegenerative biomarkers (identified in cognitive tests, abundance protein dataset, imaging data) that correlate more strongly with atrophy.

**Conclusions:**

The final aim of the integrated multimodal dataset explored with the multimodal‐oriented analysis tool is to verify the existence of endophenotypes characterizing groups of AD patients defined based on the different brain‐region atrophic component.